# New Drugs, Appliances, and Things Medical.—Addendum

**Published:** 1891-11-14

**Authors:** 


### NEW DRUGS, APPLIANCES, AND THINGS MEDICAL.

[All preparations, appliances, novelties, etc., of which a notice is desired, should be sent for The Editor, to care of The Manager, 140, Strand, London, W.C.]

### ADDENDUM.

In our recent notice of Peterman's cockroach and beetle food, it should have been stated that Messrs. Shorey are now preparing this food in such a way that it destroys red-ants as well as the vermin mentioned. This is an important feature of the food, and renders it useful to many large institutions.

